# Magnetic Solid-Phase Extraction Based on *β*-Cyclodextrins/Acrylic Acid Modified Magnetic Gelatin for Determination of Moxidectin in Milk Samples

**DOI:** 10.1155/2016/7862152

**Published:** 2016-06-29

**Authors:** Yinzhu Shang, Jing Luo, Peng Wang, Xiaoya Zhao, Cheng Ye, Shaofei Guo

**Affiliations:** Hubei Entry-Exit Inspection and Quarantine Bureau of PRC, Wuhan 430022, China

## Abstract

*β*-Cyclodextrins/acrylic acid modified magnetic gelatin was prepared and then employed as the magnetic solid-phase extraction (MSPE) sorbent for extraction of moxidectin in milk samples. Due to the rigidity of hydrophobic cavity of *β*-cyclodextrins and carboxyl groups of acrylic acid, magnetic composites are prepared to form a complex with target molecules through various kinds of chemical reactions and then showed excellent extraction performance. This method exhibits the advantages of simplicity of implementation, short extraction time (5 min), low solvent consumption, and high extraction efficiency. A rapid, simple, and effective method for the analysis of moxidectin in milk samples was established by MSPE coupled with liquid chromatography-fluorescence detection. The limit of detection was 0.1 ng·mL^−1^ and the recoveries from milk samples were in the range of 93.8%–112.5%. The relative standard deviation was not higher than 6.4%. In conclusion, magnetic solid-phase extraction is a simple and robust preconcentration technique that can be coupled to other analytical methods for the quantitative determination of target molecules in complex samples.

## 1. Introduction

The avermectins and milbemycins, which are part of the macrocyclic lactones, are potent pesticides for cow, sheep, pigs, and horses. Ivermectin (IVM), doramectin (DOR), abamectin (ABA), and eprinomectin (EPM) belong to the avermectin group, whereas MOX is one of the milbemycins and derived from the fungus* Streptomyces cyanogriseus *[1]. Ivermectin (IVM) and moxidectin (MOX) are most commonly used for animal husbandry, whereas DOR and ABA are not allowed in lactating animals [2, 3]. MOX is a potent and persistent anthelmintic, whilst oral formulations of IVM for sheep are generally less potent and persistent anthelmintics [4]. MOX is marketed as topical (pour-on) formulations for use in beef and dairy cattle. Some studies have shown that cattle treated with MOX topical pour-on achieved an advantage over the other endectocide compounds in attaining a high and persistent level of nematode control. The use of MOX during the lactation period has been correlated with a significant enhancement on the volume of milk produced. Topical formulations of MOX are currently approved for use in dairy cattle without requiring milk withdrawal time in some countries [5]. Moxidectin is active at very low doses (0.2 mg·kg^−1^) against a wide variety of nematode and arthropod parasites in cow and sheep [6]. In the European Union regulations, the maximum residue limit (MRL) value for moxidectin was 40 *μ*g·kg^−1^ in bovine and ovine milk, whereas for IVM no MRL in milk is established [7]. With the aim of protecting consumers and ensuring fair trade practices, moxidectin is monitored in food by regulatory agencies using MRL.

Moxidectin is highly lipophilic and tends to accumulate in fat tissues. The fat tissues act as a reservoir, contributing to the long term persistence in the body. Moxidectin is substantially excreted also in milk, with residues reaching up to 5% of the dose administered [8]. However, the residue of MOX in foodstuff samples could lead to undesirable risks of public health. It therefore becomes essential that the residue is strictly regulated from food safety point of view. The results showed that, after administration of MOX (200 *μ*g·kg^−1^) in goats, 5.7% of the administered dose was excreted in milk for more than 35 days after dosing [9]. Thus, it is important to develop a rapid, simple, and effective method for the determination of MOX in milk samples.

Since MOX is very potent in the antiparasitic activity and the regulated effective dose is very small, the concentration of MOX in milk is very low and simultaneously accompanied by a large number of interfering substances. Thus, sample enrichment and purification techniques are highly required prior to chromatography or mass spectrometry analysis. Protein precipitation using an organic solvent followed by solid-phase extraction (SPE) with C18 sorbents was the strategy most commonly applied for MOX analysis in foodstuff samples [10]. However, all of these reported SPE processes are time-costing because SPE consists of four steps: column preparation, sample loading, column postwash, and sample desorption [11]. In contrast, magnetic solid-phase extraction (MSPE) has received considerable attention in recent years due to its potential applications in separation science. A unique and attractive property of this technology is that magnetic adsorbents can be isolated from sample solutions by an external magnetic field [12]. Thus, suspended magnetic particles tagged with the analytes can be removed from large volume samples in a short time. The extraction procedure can be directly performed in crude samples to avoid blockage of the traditional SPE column caused by sample matrix [13].

*β*-Cyclodextrins (*β*-CD) as a novel functional monomer has attracted sufficient interest in recent years. Due to the rigidity of the hydrophobic cavity, *β*-CD unit can form a complex with the target molecules through various kinds of intermolecular interactions, such as Van der Waals force and hydrophobic interaction, which are helpful to obtain binding sites with high affinity [14]. Zhang's group successfully prepared imprinted polymers with high affinity and selectivity for erythromycin as a structural analogue of MOX using *β*-CD as the monomer [15].

In this study, magnetic/gelatin composites were prepared and modified using *β*-cyclodextrins and acrylic acid as binary functional monomers. The extraction procedure could be carried out within 5 min without extraction instrument and centrifugation. Direct contact of the composites with MOX causes direct extraction. The magnetic/gelatin composites with MOX are then separated from the solution by using an external magnetic field (magnet). Coupling with liquid chromatography-fluorescence detection (LC-FLD), a rapid, simple, and effective method was established for determination of MOX in milk samples.

## 2. Experimental

### 2.1. Materials and Instrumentations

Gelatin (from bovine skin, average molecular weight 80,000KD), acrylic acid (AA), MOX, sodium hydride (NaH), *β*-cyclodextrin (*β*-CD), and glycidilmethacrylate (GMA) were obtained from Sigma (St. Louis, MO, USA). Anhydrous ethanol, iron chloride tetrahydrate, ammonia solution, and N,N-dimethylformamide were purchased from KeMiOu Chemical Reagent Company (Tianjin, China). Ultrapure water was obtained from a Milli-R04 purification system (Millipore, Germany).

All measurements were performed using an Agilent 1200 series HPLC (Palo Alto, CA, USA) equipped with a quaternary pump, an autosampler, a thermostatic cartridge compartment, and a fluorescence detector (FLD).

### 2.2. Preparation of Acryloyl-*β*-CD

*β*-CD (3.0 g) was dissolved in 100 mL of anhydrous DMF to which NaH (0.3 g) was added. The mixture was stirred at room temperature until no gas was released, and the excessive NaH was removed by filtration. Thereafter, GMA (1.0 g) was added and formed complexes with *β*-CD through the coupling reaction accompanied with ring-opening process. Then the mixture was magnetically stirred at 90°C for 5.0 h under nitrogen protection. The complexes were obtained by washing with acetic ether several times and finally dried in a vacuum oven.

### 2.3. Preparation of Magnetic Composites

Magnetic gelatin was firstly synthesized based on the literature [16]. Briefly, gelatin (3 g) was soaked in deionized water and then dissolved at 55°C. Then, 1 mL iron chloride tetrahydrate solution was added with continuous stirring. Five milliliters of ammonia solution was then added dropwise into the solution. After 6 h, the product was magnetically collected, washed with distilled water and ethanol several times, and finally dried in a vacuum oven. Subsequently, 200 mg of magnetic gelatin was dispersed into aqueous solution and 400 *μ*L of acrylic acid (AA) was added subsequently. Then the mixture was incubated at room temperature for 12 h. Finally, magnetic gelatin, 200 mg of acryloyl-*β*-CD, and 10 mg of ammonium persulfate were gradually added and the mixture was incubated at 37°C for 12 h. The magnetic composites were washed with ethanol and deionized water, respectively, and then dried at 60°C for 24 h.

### 2.4. Determination of MOX Based on the MSPE Coupled with LC-FLD

Real milk samples were collected from a local market (Wuhan, China). An aliquot of 5 g of sample was placed into a 50 mL polypropylene tube and homogenized after adding 2.5 mL of ultrapure water. Two aliquots of 2.5 mL and one of 5 mL of acetonitrile were successively added to the samples with 10 s of mixing on a vortex between each solvent addition. After shaking for 20 min and centrifugation for 5000 rpm, the top layer was transferred to a 15 mL polypropylene centrifuge.

Subsequently, 10 mg magnetic composites were added into the extraction solution and incubated for 3 min. After magnetic separation, acetonitrile/water (9 : 1, v/v) (1.0 mL) was used to remove the sample matrix. Finally, magnetic composites were eluted with 0.5 mL of methanol/acetic acid (9 : 1, v/v) to remove MOX from the materials. The eluting fraction was collected and evaporated to dryness under a stream of N_2_. The residue was dissolved with 1 mL of acetonitrile and analyzed by LC-FLD.

For LC-FLD direct detection, extraction solution (800 *μ*L) was previously derivatized with acetic acid (40 *μ*L), trifluoroacetic anhydride (80 *μ*L), and 1-methylimidazole (80 *μ*L), at 64°C in a heating block for 20 min. After this, the derivatized solution was left at room temperature for 10 min and then analyzed by LC-FLD.

Aliquots of 20 *μ*L were analyzed by LC-FLD. All separations were performed on a SunFireTM C_18_ column (150 mm × 4.6 mm id, particle size 5 *μ*m, Waters, Milford, USA) at a flow rate of 1.0 mL·min^−1^ at room temperature. The mobile phase was acetonitrile/water solution (85 : 15, v/v). Fluorescence detector was operated at 365 nm (excitation) and 470 nm (emission).

## 3. Results and Discussion

### 3.1. Synthesis and Characterization of Magnetic Composites

**Figure 1:**
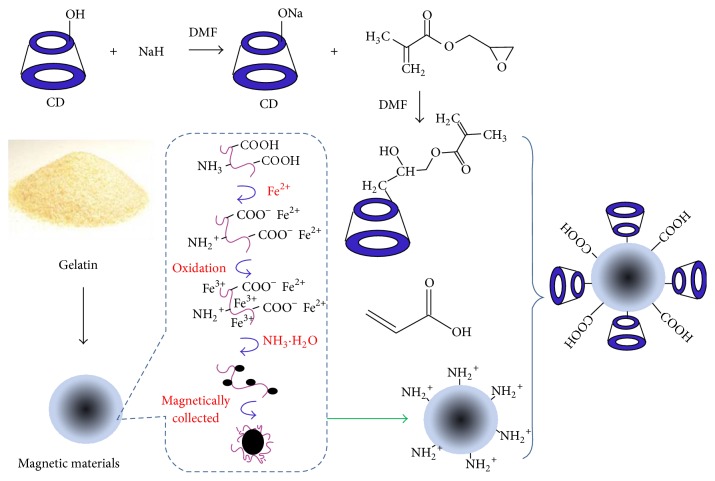
The schematic illustration of the preparation of magnetic composites.

The synthesis of magnetic composites involved preparation of magnetic gelatin, synthesis of acryloyl-*β*-CD, and modification of acryloyl-*β*-CD and acrylic acid on magnetic gelatin (Figure 1). First of all, gelatin acts as an efficient adsorbent of ionic species through polar or ionic interactions because its functional groups such as -OH, -NH_2_, and COOH act as binding sites. At low pH it bears a net negative charge on the surface in ammonia solution [16]. Thus, iron oxide nanoparticles were well distributed in the gelatin and then separated from a suspension system under an external magnetic field. Subsequently, magnetic gelatin was dispersed into an acid solution adjusted by acrylic acid (AA). At low pH solution, -NH_2_ groups of gelatin get protonated and the high adsorption capacity of acrylic acid can be expected by the electrostatic attraction between the carboxyl groups and the amino groups. Thus, double bond was introduced onto the surface of magnetic gelatin to ensure further modification of *β*-CD. Finally, acryloyl-*β*-CD and AA (1/1, w/v) were modified on the surface of magnetic gelatin using ammonium persulfate as initiator. The obtained film was beneficial for the high adsorption of MOX and then avoiding the swelling of gelatin resulted in the disintegration of magnetic gelatin.

Zeta potential measurements were performed to verify the modification on the surface of magnetic gelatin. It is shown that the zeta potential of magnetic gelatin in acid solution (pH = 5.0) was +10.14 mV, since the gelatin had a net positive charge on the surface at low pH. However, the zeta potential of AA modified magnetic gelatin was 0.78 mV due to the crosslinking of amino/carboxyl groups and decreasing of amino groups. The zeta potential of *β*-CD/AA modified magnetic gelatin was −9.21 mV, due to the carboxyl groups and hydroxyl groups of functional monomers. It was shown that the *β*-CD/AA modified films were created on the surface of magnetic gelatin.

### 3.2. Adsorption Behavior of Magnetic Composites

**Figure 2:**
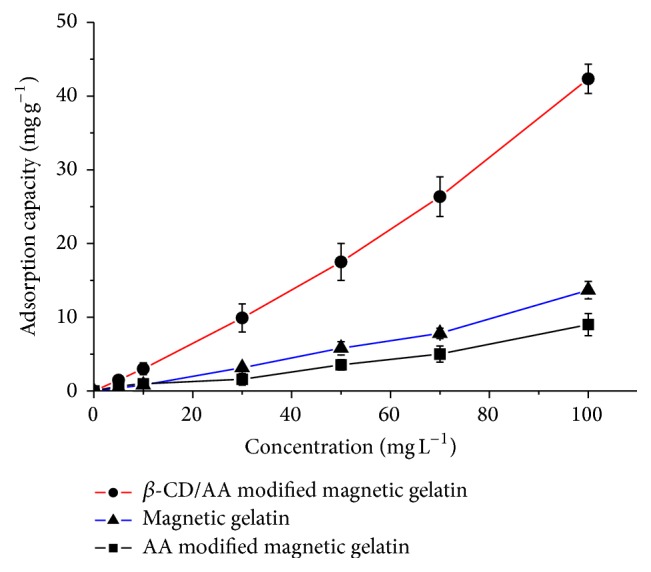
Adsorption isotherm of MOX onto the magnetic composites.

To determine the adsorption capacity of the magnetic composites for MOX, the adsorption isotherm and the corresponding Scatchard analysis were acquired.  Figure 2 shows that the amount of MOX bound to the magnetic adsorbent increased when the initial concentration of MOX increased. Compared with magnetic gelatin, the adsorption capacity of MOX on AA modified composites was lower, because the amino groups of gelatin interact with carboxyl groups of AA, which decrease the amount of functional groups. However, the adsorption capacity of MOX was the highest on the *β*-CD/AA modified magnetic composites, because of the presence of hydrophobic cavity and carboxyl groups. In the Scatchard analysis, the results indicated that *K*_*d*_ and *Q*_max_ values were calculated to be 0.003 L·mg^−1^ and 98.7 mg·g^−1^, respectively. It was shown that magnetic sorbents could be used to determine MOX in real milk samples.

### 3.3. Retention and Elution of MOX on Magnetic Composites

To evaluate the applicability of magnetic composites in the separation and enrichment of MOX in milk samples, the parameters that might affect the extraction performance were optimized. Firstly, different amounts of magnetic composites ranging from 1 mg to 15 mg were applied to extract MOX from milk samples. The results indicated that 10 mg of composites enabled almost complete recovery of MOX, and increasing the amount did not produce any significant improvement.

**Figure 3:**
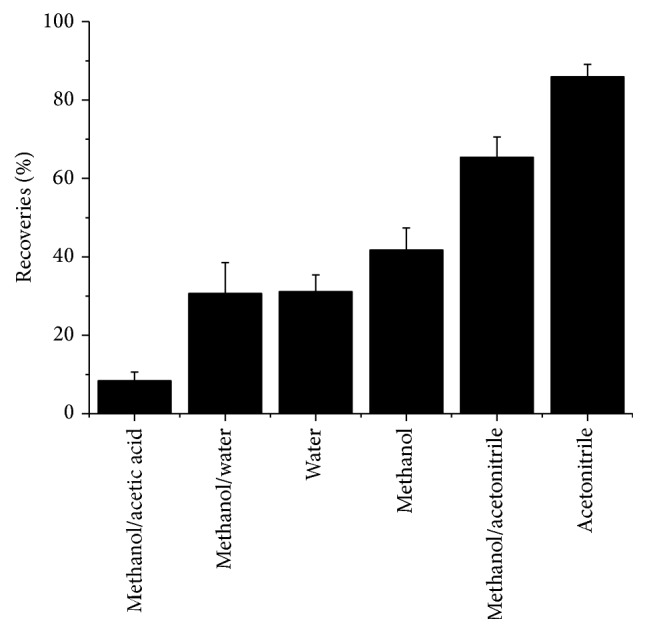
Effect of loading solution on rebinding capacity of magnetic composites for MOX.

Solvent played an important role in the MSPE procedure. Thus, different loading solvents, including water, methanol, acetonitrile, methanol/water (1 : 1, v/v), methanol/acetonitrile (1 : 1, v/v), and methanol/acetic acid (9 : 1, v/v), were used to extract 100 ng·mL^−1^ of MOX. The results are shown in Figure 3. When acetonitrile was used as the loading solvent, magnetic composites had an excellent adsorption performance for the target compound. It was also observed that methanol/acetic acid (9 : 1, v/v) was not available for extraction of the target molecule. Thus, it can be used as an eluting solution in this study and the ratio of methanol/acetic acid was optimized. The results indicated that a good eluting effect was obtained when 10% of acetic acid was mixed with the methanol solution. The volume of the eluting solvent was then studied. It was shown that 0.5 mL was enough to elute MOX completely from the magnetic composites.

Finally, extraction time profiles were conducted by increasing the vortex time from 0.5 to 5 min. It can be seen that extraction of MOX could reach equilibrium when the time is 3 min. The adsorption rapidly reached equilibrium probably because of the surface modification.

**Figure 4:**
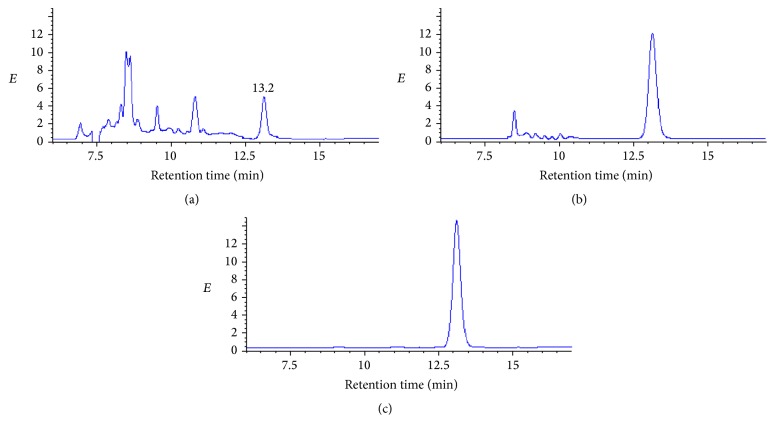
The chromatograms obtained by the spiked milk samples (100 ng·mL^−1^) with C18 SPE (a), extraction with MSPE (b), and standard solution (c).

Under the optimal protocol, milk samples spiked with MOX at 1, 10, 50, 100, and 500 ng·mL^−1^ were extracted with the magnetic composites. The extraction recoveries ranged from 83.3 to 88.9% (RSD < 8.4%).  Figure 4 shows the chromatograms obtained by the spiked sample with extraction with C18 SPE, extraction with MSPE, and standard solution. The results indicated that peak area of MOX from the spiked samples without extraction was lower than that from the standard solution, because of matrix interference in the derivatization reaction. However, efficient cleaning and high recoveries of MOX were received by MSPE procedure.

### 3.4. Analytical Performance

A simple, rapid, and low-cost analytical method based on MSPE coupled with LC-FLD was established. Under the optimal protocol, 5 mL sample solutions and 0.5 mL eluting solution were used in the extraction procedure to obtain the low limits of detection. The results indicated that linear calibration curves were obtained in the range of 1–500 ng·mL^−1^ and the linear coefficient was 0.998. It demonstrated that the proposed method could be used for quantitative analytical purposes.

Precision was evaluated by measuring intraday and interday RSDs. It was performed for the evaluation of accuracy and precision. Spiked milk sample in five replicates at different concentrations was analyzed in one day or in five different days. The intraday RSD was 4.7% and the interday RSD was 6.2% in five days. The LODs, defined as the concentrations that yielded S/N ratios greater than or equal to 3, and the LOQs, defined as the concentrations that yielded S/N ratios greater than or equal to 10, were determined through MSPE coupled with LC-FLD. In this study, the baseline noise was measured from a chromatogram of a blank sample solution. The results showed that the LOD was 0.1 ng·mL^−1^ and the LOQ was 0.5 ng·mL^−1^.

A comparative study of our developed method to other reported sample preparation procedures was performed, and the results are presented in Table 1. It can be seen that the proposed method was very simple and sensitive for the extraction of complex samples with high fat content. Compared with other studies, MSPE takes 3 min to extract MOX (100 ng·mL^−1^) in milk. It can significantly shorten the duration of the extraction process. Then magnetic composites with MOX are isolated with milk by using an external magnetic field (magnet), without the need for centrifugation or filtration. After that, just 1.5 mL washing/eluting solvent can completely elute MOX from the magnetic composites. What is more, the limit of detection is lower. Thus, this method exhibited the advantages of easy operation, shorter extraction time, low solvent consumption, and high extraction efficiency.

### 3.5. Applications in Real Samples

Real milk samples collected from a local region were used to validate the feasibility and reliability of the proposed method. MOX of spiked milk samples were extracted and determined using MSPE coupled with LC-FLD. The recoveries (*n* = 5) were in the range of 93.8%–112.5%, with RSD being not higher than 6.4%. Therefore, it was shown that the proposed method was suitable for analyzing MOX in real milk samples.

## 4. Conclusions

In summary, our approach provided a simple, rapid, and economical protocol for the determination of MOX in milk samples based on MSPE combined with LC-FLD. In this paper, *β*-CD/AA modified magnetic composites were firstly synthesized and exhibited the advantages of easy operation, shorter extraction time, low solvent consumption, and high extraction efficiency. MOX was extracted with the magnetic composites in only 5 min with only 1.5 mL eluting solvent. MOX were quantitated and confirmed by LC-FLD method. Furthermore, a large number of real milk samples were analyzed to confirm the reliability and practicality of the proposed method, and the results revealed that the method may be a promising alternative to conventional techniques for MOX in dairy products.

## Figures and Tables

**Table 1:** Comparison of the sample preparation procedures between different methods.

Matrix	Clean-up method	Volume (mL)^a^	LODs (ng·mL^−1^)	Reference
Bovine tissue	C18 SPE	16 + 8	0.1	[1]
Bovine muscle	Freeze-drying for 12 h	—	0.1	[3]
Animal liver	Alumina-N SPE	45 + 18	0.8	[8]
Milk	C18 SPE and carbon SPE	100 + 33	5	[9]
Milk	MSPE	20^b^ + 1.5	0.1	This work

^a^The volume consists of loading volume and washing/eluting volume. ^b^Because of magnetic separation and high efficiency, extraction of MOX could reach equilibrium not more than 3 min.
